# Wave-Like Spread of Ebola Zaire

**DOI:** 10.1371/journal.pbio.0030371

**Published:** 2005-10-25

**Authors:** Peter D Walsh, Roman Biek, Leslie A Real

**Affiliations:** **1** Max-Planck-Institute for Evolutionary Primatology, Leipzig, Germany,; **2** Department of Biology, Emory University, Atlanta, Georgia, United States of America; University of OxfordUnited Kingdom

## Abstract

In the past decade the Zaire strain of Ebola virus (ZEBOV) has emerged repeatedly into human populations in central Africa and caused massive die-offs of gorillas and chimpanzees. We tested the view that emergence events are independent and caused by ZEBOV variants that have been long resident at each locality. Phylogenetic analyses place the earliest known outbreak at Yambuku, Democratic Republic of Congo, very near to the root of the ZEBOV tree, suggesting that viruses causing all other known outbreaks evolved from a Yambuku-like virus after 1976. The tendency for earlier outbreaks to be directly ancestral to later outbreaks suggests that outbreaks are epidemiologically linked and may have occurred at the front of an advancing wave. While the ladder-like phylogenetic structure could also bear the signature of positive selection, our statistical power is too weak to reach a conclusion in this regard. Distances among outbreaks indicate a spread rate of about 50 km per year that remains consistent across spatial scales. Viral evolution is clocklike, and sequences show a high level of small-scale spatial structure. Genetic similarity decays with distance at roughly the same rate at all spatial scales. Our analyses suggest that ZEBOV has recently spread across the region rather than being long persistent at each outbreak locality. Controlling the impact of Ebola on wild apes and human populations may be more feasible than previously recognized.

## Abstract 2

Genetic, spatial, and temporal data reveal that the Zaire strain of Ebola virus has spread recently across the region rather than being a long-term resident in the locations where the outbreaks have occurred.

## Introduction

**Figure 1:**
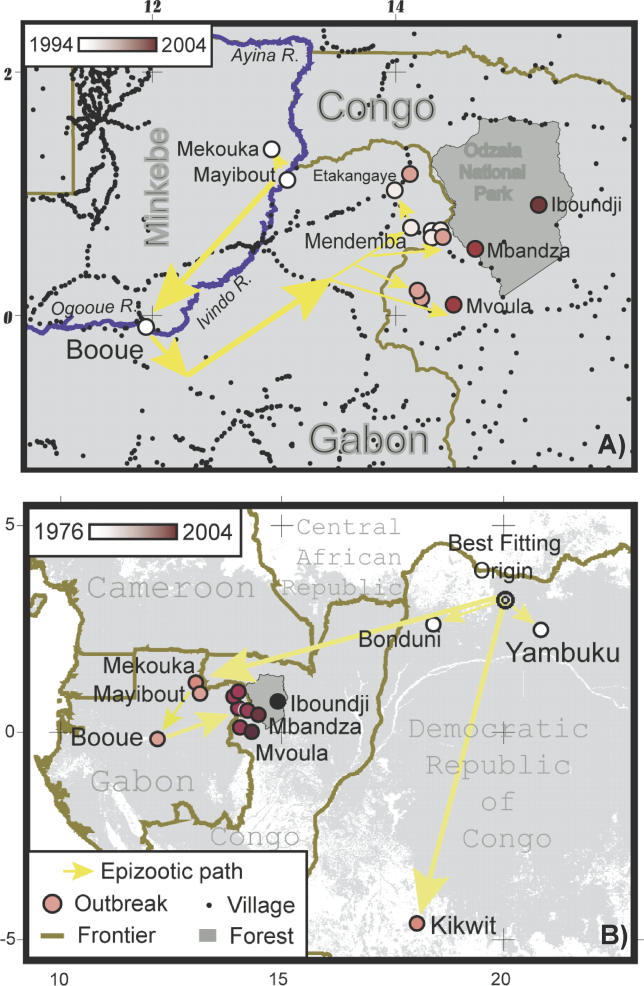
Maps of Ebola Zaire Outbreaks (A) Human outbreak locations in Gabon and Congo as reported [2]. Also shown are October 2003 human outbreak at Mbandza village and April 2004 ape die-off around Iboundji (Lokoué) Clearing in Odzala National Park. Yellow arrows represent epizootic path suggested by phylogenetic analyses. (B) Sites of all primary outbreaks of Ebola Zaire in humans documented [2,14] and the epizootic path suggested by the spatio-temporal pattern of outbreaks (yellow arrows). Best fitting origin found through ML search for the spatial location that produced the strongest correlation between outbreak date and geographic distance from the origin. ML search based on the correlation between patristic genetic distance and spatial separation between outbreaks places the epizootic pivot point just southeast of Booué. In both figures, shading of circles is proportional to time after first outbreak in series.

In the past decade, the highly virulent Zaire strain of Ebola virus (ZEBOV) has repeatedly emerged into rural human populations in Gabon and Republic of Congo [1,2] (Figure 1). Compelling genetic evidence [2] suggests that ZEBOV entered human populations when people handled infected carcasses of western gorillas *(Gorilla gorilla)* and common chimpanzees *(Pan troglodyte*s*)* during massive ape die-offs [3,4]. The risk of new ZEBOV outbreaks in the two countries poses a continuing threat to humans as well as to the largest remaining gorilla and chimpanzee populations in the world.

It seems highly unlikely that ZEBOV has caused similarly damaging ape die-offs in Gabon and Congo during the past century. Ape reproductive rates are so low that recovery from population reductions as dramatic as recently caused by ZEBOV would take 75 years or more [4]. Thus, if large ape die-offs had occurred in the past half-century, one would expect to find large zones of low ape density. Extensive surveys conducted during the 1980s and early 1990s [5–10] showed no such evidence. This observation is consistent with the fact that no human outbreaks of ZEBOV were recognized in Gabon or Congo before 1994. The big question is, thus: Why has ZEBOV now emerged so explosively?

There are two contrasting answers to this question. First, many authors have either assumed [11–14,] or concluded [2,15–19] that ZEBOV has long been present in the region and that its emergence is due to an increase in the rate at which human or non-human apes come into contact with some yet-to-be-identified reservoir host. Both habitat disturbance [1,16] and climatic factors [2,20,21] have been proposed as triggers for ZEBOV emergence. The alternative, which so far has received little attention, is that the virus we know as ZEBOV has actually spread only recently to each outbreak site.

While the history of ZEBOV has so far remained elusive, examples from many other viruses show that the spatio-temporal dynamics of a virus are reflected in its phylogenetic structure [22–24]. Viruses that have long been maintained within a single host population, for example, tend to have a high diversity of genetic lineages, especially if they are subject to little or no selection at the host population level. Given sufficient levels of dispersal, related genotypes may be widely distributed and will show little spatial clustering. Hepatitis C virus, for instance, is considered to have a long association with humans, but has a number of strains with worldwide distribution, probably due to inadvertent infections resulting from medical interventions at a global scale [25].

In contrast, restricted genetic diversity and rapid turnover of genotypes are hallmarks of viruses that are either spreading or are subject to continuous positive selection. Fox rabies and influenza A are typical examples, respectively [22,23]. Although, in either case, phylogenies exhibit a characteristic ladder-like pattern, the underlying mechanism (genetic drift in the former, selection in the latter) is fundamentally different and should leave distinguishable signatures in the spatio-temporal distribution and genetic substitution patterns of the virus. Given these general principles, our aim for the present study was to use a combination of genetic, spatial, and temporal data to discriminate between the two hypotheses for ZEBOV emergence (i.e. long term, local persistence versus recent spread).

Genetic information for testing these hypotheses is available from gene sequences sampled from human outbreaks. If ZEBOV has been persistent at localities across the region for hundreds or thousands of years, then the virus should have diverged into a number of distinct genetic lineages whose most recent common ancestor (MRCA) long pre-dates the first recognized ZEBOV outbreak in 1976 at Yambuku, Democratic Republic of Congo (DRC). Furthermore, outbreaks subsequent to Yambuku could equally have been caused by more ancestral or more recently derived lineages. On the other hand, if ZEBOV has spread through the region only recently, all viruses sampled should be descendants of the same genetic lineage with an MRCA close to the 1976 sequence from Yambuku. Along a given spatial trajectory, genotypes involved in more recent outbreaks should be more or less direct descendants of viruses found during previous outbreaks (creating the characteristic ladder-like pattern). Successive outbreaks should also be progressively more divergent from the MRCA. The only reasonable scenario under which such a pattern would be expected from a long-resident virus is one of continuous selection for new virus variants. However, identical selection pressures would have to apply over much of the geographic range of the virus, and movement throughout the range would have to be high for the same selected variants to occur in different localities (e.g., influenza [23]).

A second source of information lies in the spatio-temporal pattern of ZEBOV emergence. Under either hypothesis, local transmission events during a given outbreak might result in new cases appearing further and further away from the outbreak origin. However, if outbreaks are truly independent emergences from a persistent, widely distributed ZEBOV population, no spatial trend should be apparent in the locations of different outbreaks over the entire period since 1976. In contrast, if ZEBOV has spread from a mid-1970s origin near Yambuku, then new outbreaks should move further and further away from Yambuku as time passes. If ZEBOV is transmitted through some sort of local contact process, then the rate of spread should be consistent across spatial scales (Protocol S1). If the spreading wave has made changes in direction, then outbreak date should be correlated with geographic distance along the invasion corridor rather than simply with straight-line distance from Yambuku.

A third class of information lies in the spatial structure of virus genotypes. Outbreaks at the front of a spreading wave should show a correlation between genetic and spatial distance that is detectable at different spatial scales (Protocol S1). Changes in the direction of spread might weaken such isolation by distance at large scales, but correlation strength would remain high if spatial distance was measured from the origin of the wave and along the putative path of spread. The development of strong spatial structuring would also be possible in a long-resident virus, but not necessarily, as high levels of gene flow would tend to spatially randomize genotypes [26]. Thus, the absence of spatial structuring would argue against spread, but not necessarily against local persistence.

We tested this series of predictions regarding local persistence or recent spread of ZEBOV by analyzing data on the spatio-temporal pattern of outbreaks together with glycoprotein (GP) gene sequences collected from human outbreaks. We found the data to be inconsistent with the idea that the ZEBOV outbreaks of the past 30 years are caused by a virus that has been a long-term resident at each site. Instead, all our results are concordant with the hypothesis of a recent ZEBOV wave that spread through the area in a relatively consistent and predictable manner.

## Results

### Phylogenetic Structure and Selection Patterns

**Figure 2:**
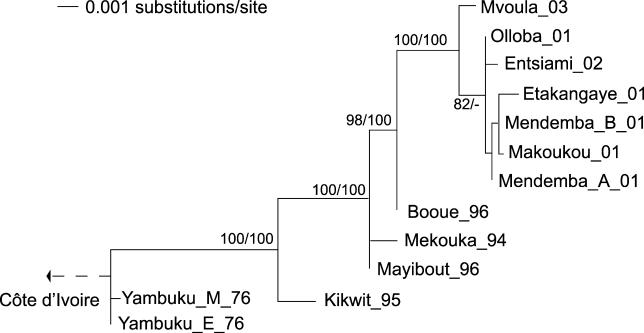
ML Tree of Full-Length (> 2,000 bp) ZEBOV-GP Sequences Tree was found in Paup* and rooted in a separate analysis using ICEBOV as an out-group. The latter analysis excluded a 576-bp variable region for which alignment with ZEBOV was uncertain (see Materials and Methods). Numbers next to branches indicate percent support based on 1,000 bootstrap replicates and posterior probabilities obtained in a molecular clock-based analysis in program BEAST (only values > 70% are shown).

Maximum likelihood (ML) and Bayesian phylogenetic estimation approaches produced highly similar phylogenetic trees in which only one major lineage could be distinguished (Figure 2). All of the major structural features showed high statistical support. Both approaches placed the earliest outbreak (Yambuku,1976) very near the tree root (which estimates the MRCA), implying that ZEBOV sequences obtained at all other localities evolved from a virus very similar to Yambuku sometime after 1976.

Both trees also exhibited a series of ancestor–descendant relationships between outbreak localities (i.e., Yambuku→Mayibout, Mayibout→Booué, Booué→Mendemba) that closely mirrored the time sequence of ZEBOV outbreaks, with the most recent outbreaks falling furthest from the tree root. The tendency for new outbreaks to be directly descendent from immediately preceding outbreaks implies that outbreaks have occurred only in newly infected areas: either at the front of a narrow, advancing wave or through a series of long jumps in which each outbreak was seeded by the previous one. The rate of nucleotide substitution thereby remains fairly constant through time, as a molecular clock model could not be rejected (Table S1).

Because the observed rapid turnover of viral genotypes could potentially be the consequence of selection for new variants, we tested for positive selection in two ways. First, we examined the ratio of non-synonymous to synonymous substitutions (d_N_/d_S_) along tree branches. A model distinguishing different d_N_/d_S_ for internal and tip branches did not fit any better than a model with one ratio applied to the entire tree (*p* = 0.395; Table S2), indicating that there was no relative increase in d_N_ associated with branches that gave rise to future lineages. Such an increase would be expected if positive selection were a major driving force behind the rapid turnover of virus genotypes [27]. But, given the small overall number of mutations on the tree, our statistical power to detect such an effect was low, especially if only a small number of sites were subject to positive selection.

In our second analysis, we tested whether individual sites showed evidence of selection. A model of nearly neutral evolution was rejected in favor of a model accounting for sites under positive selection (*p* = 0.009; Table S2). However, only one codon site (amino acid position 370) had a posterior probability greater than 95% of being under positive selection in a subsequent Bayesian assignment. This site appeared to have undergone three changes back and forth between isoleucine and methionine (Figure S1). A number of sites experienced a single amino acid change, many of which fell on internal branches. While these results are consistent with positive selection playing a role in ZEBOV evolution, they are inconclusive as to whether the specific amino acid replacements we observed are truly due to Darwinian selection or merely represent neutral evolution.

### Spatio-Temporal Structure of Outbreaks

**Figure 3:**
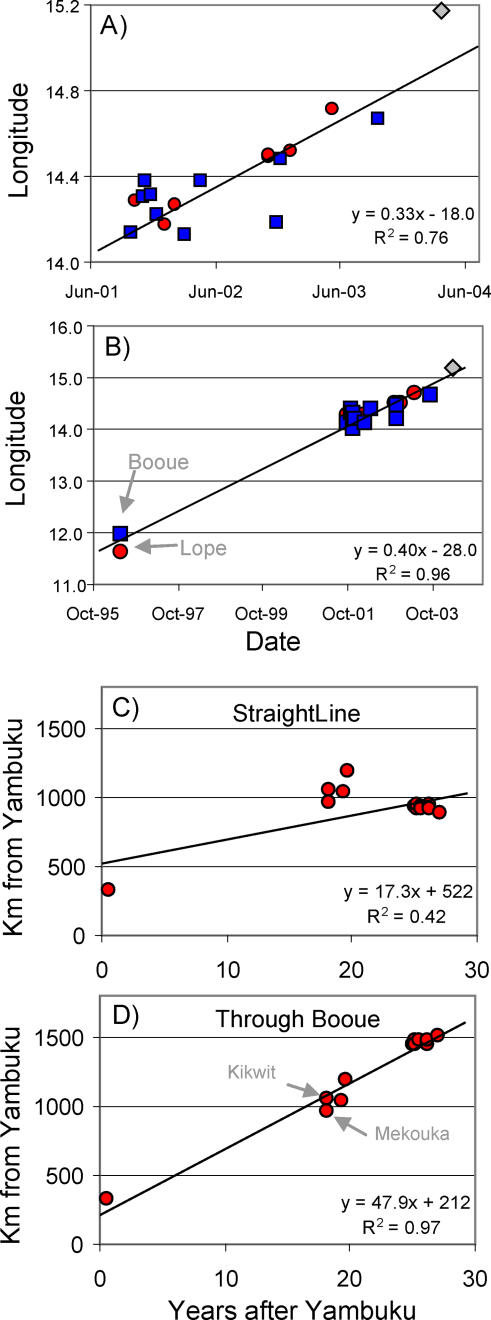
Spatial Spread of ZEBOV (A) Relationship between date and longitude of outbreaks in Gabon-Congo border area. Blue squares, human outbreaks [2] and 2003 outbreak at Mbandza village; red circles, animal carcasses testing positive for Ebola [18]; gray diamonds, ape die-off at Ibounji\Lokoue clearing. Regression line is for pooled data. Analyzed separately, human outbreaks and Ebola+ animal carcasses both show significant correlations between longitude and date (human outbreaks *n* = 12, R^2^ = 0.48, *p* = 0.01; animal carcasses *n* = 13, R^2^ = 0.91, *p* < 0.001). (B) Added are 1996 human outbreak at Booué and Ebola+ chimpanzee carcass from nearby Lope [1]. The lack of reported human outbreaks between 1996 and 2001 may simply reflect the extremely low village density between Booué and Mendemba (Figure 1). (C) Time after Yambuku versus straight line distance from Yambuku to all subsequent human outbreaks, including [2,14] and Mbandza village (R^2^ = 0.42, *n* = 17, *p* = 0.005). (D) Same as (C) but with distance from Yambuku to the recent Gabon-Congo border outbreaks measured as passing through Booué (R^2^ = 0.97,*n* =17, *p* < 0.001). All figures include outbreak sites cited [2,14] for which no ZEBOV-GP sequences were publicly available.

ZEBOV outbreaks showed a distinct spatio-temporal pattern, both over the entire period since 1976 and during shorter time intervals. For example, between 2001–2004 in the Gabon-Congo border area, both human outbreaks and animal carcasses that tested positive for Ebola independently showed statistically significant patterns of eastward spread (Figure 3A). Furthermore, the eastward spread rate estimated for the 2001–2004 period (46.1 km per year) changed little if the 1996 human outbreak at Booué and a nearby Ebola-positive chimpanzee carcass were added to the analysis (47.6 km per year; Figure 3B). This rate consistency over a large temporal interval is concordant with a single spread process, from Booué eastward through the Gabon-Congo border area.

The pattern of spread from west to east does not continue with the outbreaks preceding Booué. However, the ancestor–descendant relationships in the ZEBOV phylogeny (Figure 2) suggest a coherent spread pattern in the period preceding the Booué outbreak. The position of Yambuku near the tree root suggests that the ZEBOV spread originated somewhere near Yambuku in about 1976 and continued both south to Kikwit and west to Booué (Figure 1B). This hypothesis was supported by a ML search for the epizootic origin that maximized the correlation between geographic distance from the origin and time after the origin. This search chose a February 1973 origin just northwest of Yambuku (Figure 1B). The phylogenetic position of the Booué sequence as a direct ancestor to all of the 2001–2003 outbreaks (Figure 2) suggests that the western front then turned eastward toward the Gabon-Congo border. This abrupt change of direction may have been caused by natural features such as rivers, as frequently observed in spreading pathogens [28–30], and was suspected prior to any genetic data becoming available (see Discussion).

The hypothesis of a pivot point at Booué was strongly supported by the observed relationship between geographic distance from the putative origin at Yambuku and time after Yambuku. If geographic distances from all other outbreaks to Yambuku were measured in a straight line, then the relationship was significant but relatively weak (Figure 3C). However, if geographic distances to the Gabon-Congo border outbreaks were routed through Booué, correlation strength increased dramatically (Figure 3D). The great strength of this relationship was not due to a single outlier point, as all of the major legs of the putative epizootic path showed similar spread rates (Yambuku→Kikwit = 51.7 km per year, Yambuku→Mekouka = 56.9 km per year, Booué→Mendemba = 48.5 km per year, Mendemba→Iboundji = 47.9 km per year).

### Spatial Structure of Genotypes

**Figure 4:**
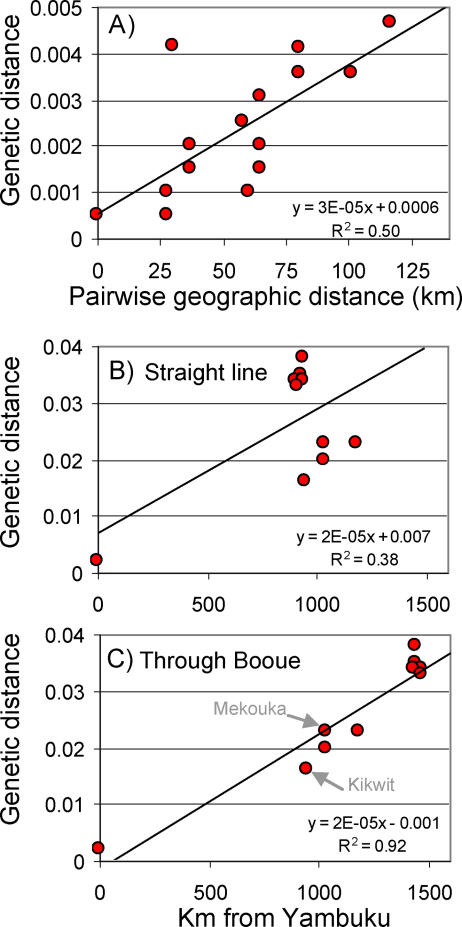
Correlation between Geographic Distance and Patristic Genetic Distance (A) ML genetic distances (substitutions per nucleotide site) plotted as function of geographic distance separating pairs of outbreak sites for the six full-length, georeferenced sequences sampled by Leroy et al. (R^2^ = 0.70, Mantel test *p* = 0.002). Makokou and Yembelengoye sequences excluded because of unknown spatial origin of case and partial sequence, respectively (Protocol S3). (B) Correlation between straight line distance from the initial ZEBOV outbreak site at Yambuku and patristic genetic distance to Yambuku for all available georeferenced, full-length sequences (R^2^ = 0.38 , *n* = 11, *p* = 0.040). (C) Same as (B) but with geographic distances to the recent Gabon-Congo border outbreaks measured as passing through Booué (R^2^ = 0.92 , *n* = 11, *p* < 0.001).

At the local as well as the regional scale, spatial structure was evident in the distribution of ZEBOV genotypes. For instance, the 2001–2003 outbreaks on the Gabon-Congo border showed a clear pattern of decreasing genetic similarity with increasing geographic distances (Figure 4A). The tight spatial structuring of genotypes at this relatively small scale fits the notion that ZEBOV transmission is a local contact process involving short movements of a few kilometers or less [26], a conclusion concordant with the observed consistency in the time rate of epizootic spread (Figure 3A and 3B).

**Figure 5:**
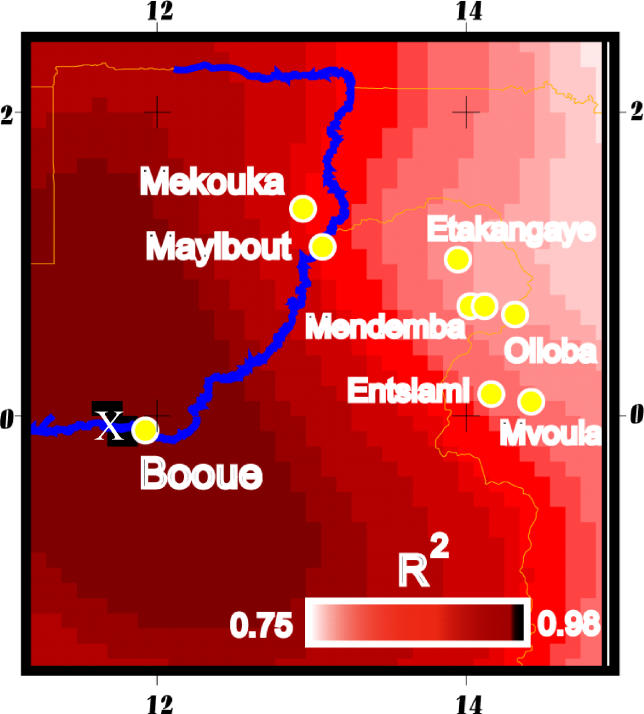
Epizootic Pivot Point Shading of each grid cell indicates the strength of correlation (R^2^) between geographic distance and patristic genetic distance when that grid cell (rather than Booué) is used as the epizootic pivot point. The position of the best fitting pivot point (shown with a white X) along the Ogooue River (blue line) is consistent with a river crossing near Booué, with subsequent movement east toward the Mendemba area. It is not consistent with gene flow directly between the other mid-1990s outbreak localities (Mekouka and Mayibout) and Mendemba.

Geographic structuring of genotypes was also evident at higher spatial scales. The correlation between geographic distance to Yambuku and genetic divergence from Yambuku was only weak. However, as with the spatio-temporal analysis, a striking improvement in model fit was observed when geographic distances were routed through Booué (Figure 4B and 4C). A ML search for an epizootic pivot point that maximized the correlation between geographic distance and genetic divergences chose a pivot point just west of Booué (Figure 5).

## Discussion

Our results clearly challenge the belief that ZEBOV has been persistently present for a long time at the outbreak sites in Gabon and Congo. First, our phylogenetic results imply that all known ZEBOV emergences occurring after Yambuku in 1976 were caused by direct and closely related descendents of a Yambuku-like virus. The descent of all known ZEBOV viruses from a very recent common ancestor is clearly inconsistent with the notion that they have long been evolving independently and in situ.

Second, a similar ancestor–descendent relationship connects the outbreaks of the mid-1990s to those of 2001–2004 (Figure 2). This replacement of virus over time and space by closely related but progressively more divergent genotypes is typically observed under spatial spread or continuous positive selection. Although our analyses were consistent with some codon sites being under positive selection, statistical power was too weak to reliably identify such sites. Fit of the molecular clock suggests that many of the observed substitutions are effectively neutral, so that the number of positively selected sites may be small. More importantly, spread and adaptation are not mutually exclusive. In fact, the very recent descent of all ZEBOV variants from a Yambuku-like common ancestor necessarily implies relatively rapid spread of variants across a large range. Thus, whether or not the ladder-like structure of the ZEBOV tree bears a signature of positive selection, it is much more consistent with recent spread than with independent evolution at each outbreak locality.

**Figure 6:**
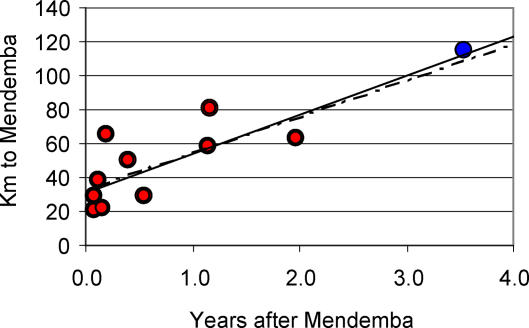
Spatial Spread from 2001–2005 Distance of each human outbreak site from the initial outbreak at Mendemba village, Gabon, plotted as a function of time after the Mendemba outbreak. Dashed regression line uses only outbreaks from 2001–2003 (R^2^ = 0.43, *p* = 0.04). Solid regression line includes May 2005 outbreak at Etoumbi village (R^2^ = 0.73, *p* < 0.001).

Third, we found a general correlation between when new ZEBOV cases were observed and their geographical distance to previous cases. Importantly, this relationship and the corresponding rate of spread of about 50 km per year remained consistent over multiple spatial scales. The low *p* values in our correlation analyses indicate that observing such a pattern by chance, as the hypothesis of long-term presence of ZEBOV in the area would require, would be highly unlikely. A recent ZEBOV outbreak in May 2005 at Etoumbi village, which occurred after this paper was submitted for review, further provided an opportunity to test our model. Reassuringly, the spread rate from 2001–2003 did an excellent job of predicting the Etoumbi outbreak, given its distance from the 2001 outbreak at Mendemba (Figure 6).

Fourth, we identified a pattern of constantly increasing genetic divergence among virus genotypes with increasing geographic distance. As pointed out initially, a roughly linear relationship between genetic divergence and geographic separation is an expected outcome under spatial spread, particularly if spread has occurred along a relatively narrow front (Protocol S1). Although isolation by distance itself could also be found among locally resident viruses, such a scenario would be inconsistent with any of the previous results, including the dramatically improved fit of the spatial-genetic correlation when routing distances through Booué.

Taken together, our results clearly point to the conclusion that ZEBOV has gradually spread across central Africa from an origin near Yambuku in the mid-1970s. Under this scenario, the distinct phylogenetic tree structure, the strong correlation between outbreak date and distance from Yambuku, and the correlation between genetic and geographic distances can be interpreted as the outcome of a consistently moving wave of ZEBOV infection.

The large-scale spatial correlations we identified were particularly strong under the assumption that the ZEBOV wave changed direction at Booué. This hypothesis may seem ad hoc but was actually posed by one of the authors (PDW) in a paper published a year before genetic data from the Gabon-Congo border region became available [4]. Transect surveys and numerous reports from local villagers had suggested that the second largest river system in equatorial Africa (the Ogooue-Ivindo-Ayina) had largely contained the 1994–1996 outbreaks in the Minkebe region of northern Gabon [3,4]. An Ebola-positive chimpanzee was then found south of the river near Booué in 1996 [1], and subsequent surveys revealed suspiciously low ape densities southeast of Booué. Thus, all of the genetic correlation analyses we report here represent independent confirmation of an a priori hypothesis of spread from Booué, posed before genetic data were available. Likewise, the phylogenetic analysis, which identified the Booué virus sequence as the direct ancestor of all viruses observed later near the Gabon-Congo border, also indicates that Booué forms an epidemiologic link between previous and subsequent outbreaks. The effect of major rivers in channeling spread is well documented for other diseases in natural populations [28–30].

Whether ZEBOV was resident (but undetected) in the central African forest block before the mid-1970s, or is an invader from outside the region remains unclear. Blood samples taken from both human [11] and non-human primates [17] suggest that some filovirus was already present in western equatorial Africa before the mid-1990s ape die-offs. Unfortunately, the serological tests employed were not specific to ZEBOV [31]. Therefore, it is impossible to tell whether these positive results were caused by a virus with a very recent common ancestor of the lineage we know as ZEBOV or by some more distantly related virus that is cross-reactive. The co-occurrence of both moderately high seropositivity and high ape densities at some sampling localities argues that the assayed virus was not highly virulent. ZEBOV has caused such high mortality rates in recent ape outbreaks that by the time these populations recover to high density (if they recover), individuals born after the outbreaks will greatly outnumber seropositive survivors (if any are still alive). Thus, moderate to high levels of seropositivity in ape populations are not consistent with high virulence. The absence of large human outbreaks in western equatorial Africa before the mid-1990s is consistent with a non-virulent virus, although the possibility that smaller outbreaks occurred but were not recognized cannot be excluded.

The high rate of positive results in past serological surveys may explain why previous authors appear not to have seriously considered the possibility of recent ZEBOV spread. Apart from the serological results, the other major argument for long-term persistence at each locality has involved the mutational stability of ZEBOV-GP. The absence of mutations within several closely monitored human transmission chains has been used to argue that ZEBOV-GP evolves too slowly for a wildlife epizootic lasting only a few years to have generated the sequence variation observed in the recent Gabon-Congo border outbreaks [2,18]. However, a formal statistical power analysis shows that the number of human cases involved in the cited transmission chains was far too small to reach this conclusion (Protocol S2). In fact, our molecular clock analyses showed that ZEBOV evolves at a rate comparable to other RNA viruses, about 8 × 10^−4^ substitutions per site per year (Table S3) and that the MRCA of the Gabon-Congo border outbreaks occurred in 1999 (CI = 1998–2000), well after the 1996 Booué outbreak. Thus, the genetic stability noted between ZEBOV outbreaks appears to be the consequence of short time separation rather than slow evolution.

Although our results strongly support the hypothesis that ZEBOV spread recently to the outbreak sites in Gabon and Congo, it is still unclear through which reservoir host(s) ZEBOV spread occurred. Spread might have taken place through transmission within some wildlife reservoir endemic to the region or through the wave-like invasion of an infected reservoir. Whatever the reservoir species or group of species, the striking constancy in the rates of ZEBOV spread and evolution suggests either that its distribution and abundance are fairly uniform throughout the affected area, or that its range has been expanding at a uniform rate. At the same time, we found that the large-scale pattern of spread is well represented as one-dimensional, which contradicts expectations for a radiating wave in a uniformly distributed host. As pointed out before, we suspect that the channeling effect of rivers may be responsible for this pattern. The large time gaps between human outbreaks may simply reflect the fact that much of the proposed epizootic path is very lightly inhabited by humans. For example, the 1996 outbreak site at Booué and the 2001 outbreak site at Mendemba are separated by 250 km of forest crossed by a single road, along which lie only a handful of small villages.

The conclusion that ZEBOV has recently spread further begs the question of whether this spread was triggered by some ecological change (perhaps anthropogenic in origin), by some change in the virus itself (for example, a mutation to higher virulence), or simply by some stochastic event. Answering these questions remains a challenge for future research. To what extent ZEBOV transmission between apes plays a role in either ZEBOV spatial spread or ape die-offs also remain open questions.

Our results also warrant a re-evaluation of the potential for Ebola control. The consistent rate of Ebola spread suggests that control efforts may not need to encompass the entire region, but could be concentrated directly ahead of the advancing wave. Knowledge of the future path of spread could be used to strategically allocate the delivery of an Ebola vaccine [32–34] (cf. rabies [35]) when a successful vaccine is developed.

If the past spread rate of about 50 km per year continues in the current direction, Ebola Zaire should hit the populated areas north and east of Odzala National Park within the next one to two years. Most of the handful of parks still containing populations of gorillas large enough to be viable in the long term might be reached within three to six years. Saving these viable ape populations should be a top priority.

## Materials and Methods

### Phylogenetic estimation

Our phylogenetic analyses included 13 of the 14 published ZEBOV-GP gene sequences, including sequences from the Gabon-Congo border outbreaks of 2001–2003. A. Sanchez (Centers for Disease Control and Prevention) kindly provided us with a sequence from the 1996 outbreak at Mayibout, Gabon. As the out-group for our analyses, we used the sequence from the 1994 Ebola outbreak at Tai Forest, Cote d'Ivoire (ICEBOV, the strain genetically closest to ZEBOV).

Much of the information for estimating time rates of divergence from the ZEBOV root lies in a hyper-variable region in the middle of GP [36]. This region is highly divergent from the closest out-group, ICEBOV [37], causing problems of saturation and precluding accurate alignments even at the amino acid level. Therefore, we initially trimmed the hyper-variable region (sites 925–1,500) and used ICEBOV as the out-group to estimate the topological root of ZEBOV based on the remaining 1,473 base pairs (bp). A ML tree using all available ZEBOV sequences greater than 2,000 bp was found in a heuristic search under a GTR+G model in Paup*4.0b10 [38]. Keeping the resulting tree root, we then excluded ICEBOV and used the full-length sequences (2,049 bp) to re-estimate tree topology (which remained virtually the same) and branch lengths for ZEBOV only. Selection of the evolutionary models and model parameters was done in Modeltest 3.6 [39] using recommendations by Posada and Buckley [40]. In parallel, we used the dated full-length sequences in a Bayesian coalescent analysis that simultaneously estimated tree topology and the rate of clocklike divergence from the root but did not require an out-group (see next paragraph).

We estimated the evolutionary rate of ZEBOV by taking advantage of the temporal spread of sequence sampling, spanning almost three decades. Based on the rooted ML tree obtained, program TipDate [41] was used to estimate the ML rate of evolution under the “single rate dated tips” model. Fit of the “single rate dated tips” model was assessed against a model with unconstrained branch lengths (i.e., different rates for each branch) and a single rate model that did not take sampling dates into account. In addition to the ML estimates, Bayesian coalescent-based estimates of the evolutionary rate and tree topology of ZEBOV under a “single rate dated tips” model were obtained in the program BEAST [42]. Two independent runs were performed with 20 million states each, of which the first 2 million were removed as burn-in. Along with the two parameters of interest (rate and topology), the program yielded estimates of the transition/transversion ratio and proportion of invariant sites. From the states visited, 9,000 trees (one every 4,000 states) were used to compute posterior probability frequencies of support for individual nodes. Besides strengthening support for most of the nodes identified in the ML analysis, this analysis independently placed the Yambuku sequences at the root of the ZEBOV tree. Varying prior assumptions about the demographic history of ZEBOV (exponential growth and decline instead of constant population size) had virtually no effect on the results (unpublished data). Further information on methods and our choice of sequences can be found in Protocol S3.

### Testing for positive selection

Only 29 inferred d_S_ and 39 inferred d_N_ were found in the ZEBOV-GP phylogeny. Although the small number of changes clearly limited our statistical power, we attempted to characterize selection patterns for the GP gene using the program CodeML [43]. First, we tested for evidence of positive selection on particular branches. If replacement of viral lineages over space and time were due to positive selection, non-synonymous changes should be found particularly on the internal branches of the tree because these branches represent lineages that have spread successfully, potentially due to a fitness advantage [44,45]. By the same argument, relative rates of d_N_ to d_S_ should be higher in the case of positive selection for internal branches compared with external branches, which left no descendents within our sample. Therefore, we estimated different d_N_/d_S_ ratios for internal branches and branches connected to tree tips and compared this with a model with a single ratio for all branches. Secondly, we tested for the presence of particular codon sites under positive selection [46], followed by the identification of such sites using an empirical Bayes procedure [47]. For all tests, relative fit of nested models was determined by using likelihood ratio testing. Finally, we used ancestral reconstruction to map putative amino acid substitutions onto the phylogeny.

### Estimating epizootic origin

To find a putative epizootic origin, we posited that an epizootic wave started somewhere in central Africa sometime before the 1976 Yambuku outbreak, spread outward at a constant rate, then made a change of direction at Booué. Under these assumptions, the spatial distance (d_i_) of outbreak location i from a putative point of origin should be a linear function of time (t_i_) since the outbreak at the putative origin



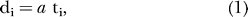



with distances to the Gabon-Congo border outbreaks measured through Booué. We estimated the origin by searching for a date, location, and value of the slope parameter *a* that minimized the squared differences between the predicted distance and the observed distance summed over all outbreak locations (including Yambuku). The best fitting date and location was in the forest–savannah transition zone just to the north and west of Yambuku (4.0°N, 21.6°E) in February 1973 (Figure 1B). The coefficient of determination for this best fitting origin (R^2^ = 0.99; *n* = 18; *p* < 0.001) was only marginally better than the coefficient of determination achieved using Yambuku as the origin (Figure 3C).

### Estimating epizootic pivot point

To search for the best fitting pivot point for the epizootic, we performed a Pearson product-moment correlation between the geographic distance of each outbreak site along the putative epizootic path from Yambuku and the patristic genetic distance of that outbreak site from Yambuku. However, instead of routing geographic distances from Yambuku to the recent Mendemba series of outbreaks through Booué (as in our other analyses), we cut the region into a 0.1° (about 11 km) grid and routed distances through the midpoint of each cell on the grid. The cell that produced the strongest correlation between geographic and genetic distance from Yambuku (plotted in Figure 5 as the coefficient of determination, R^2^) was taken to be the ML pivot point. We excluded Kikwit from this analysis because our phylogenetic analyses suggested that it was a distinct lineage that had diverged from the Gabon-Congo lineage at some unknown location south and west of their common ancestor Yambuku. Because we had no a priori hypothesis about where in space this divergence occurred, we had no means of calculating the spatial distances between the Gabon-Congo outbreaks and Kikwit.

## Supporting Information

Protocol S1 Simulating Spatial Spread (333 KB PDF).

Protocol S2 Statistical Power Analysis (173 KB PDF).

Protocol S3 Phylogenetic Estimation (228 KB PDF).

Figure S1 Selection Analysis Distribution of inferred amino acid changes on the ZEBOV phylogeny. Substitutions were estimated using ancestral reconstruction in program CodeML. Codon site 370, the only site identified as being under positive selection, is shown in blue.
(123 KB DOC)

Table S1 Support For the Molecular Clock in ZEBOV (100 KB PDF).

Table S2 Assessing Fit of Selection Models [46] to the ZEBOV Glycoprotein Data Using Likelihood Ratio Testing (100 KB PDF).

Table S3 Evolutionary Rate Estimates for ZEBOV Obtained under ML in TipDate and under a Bayesian Framework Using Markov Chain Monte Carlo Integration in BEAST (89 KB PDF).

### Accession Numbers

The GenBank (http://www.ncbi.nlm.nih.gov/GenBank) accession numbers for the gene sequences discussed in this paper are: the Booué Gabon outbreak of 1996 (AY058898); the Gabon-Congo border outbreaks of 2001–2003 (Ektakangaye, AY526100; Entsiami, AY526102; Makoukou, AY526101; Mendemba A, AY526105; Mvoula, Ay526104; Olloba, AY526099); the Kikwit, DRC outbreak of 1995 (U28077.1); the Mekouka Gabon outbreak of 1994 (U77384.1); the Tai Forest, Cote d' Ivoire outbreak of 1996 (U28006); and the Yambuku, DRC outbreak of 1976 (Eckron 76 [Yambuku-E], U81161.1; Mayinga [Yambuka-M], U231887.1).

## Abbreviations

bp: base pair
d_N_: non-synonymous substitution
d_S_: synonymous substitution
GP: glycoprotein
ICEBOV: Ebola Cote D' Ivoire
ML: maximum likelihood
MRCA: most recent common ancestor
ZEBOV: Zaire strain of Ebola virus

## References

[pbio-0030371-b01] Georges AJ, Leroy EM, Renaut AA, Benissan CT, Nabias RJ (1999). Ebola hemorrhagic fever outbreaks in Gabon, 1994–1997: Epidemiologic and health control issues. J Infect Dis.

[pbio-0030371-b02] Leroy EM, Rouquet P, Formenty P, Souquiere S, Kilbourne A (2004). Multiple Ebola virus transmission events and rapid decline of central African wildlife. Science.

[pbio-0030371-b03] Huijbregts B, De Wachter P, Obiang LSN, Akou ME (2003). Ebola and the decline of gorilla *Gorilla gorilla* and chimpanzee *Pan troglodytes* populations in Minkebe Forest, north-eastern Gabon. Oryx.

[pbio-0030371-b04] Walsh PD, Abernethy KA, Bermejo M, Beyers R, De Wachter P (2003). Catastrophic ape decline in western equatorial Africa. Nature.

[pbio-0030371-b05] Tutin CEG, Fernandez M (1984). Nationwide census of gorilla *(Gorilla gorilla)* and chimpanzee *(Pan troglodytes)* populations in Gabon. Am J Primatol.

[pbio-0030371-b06] Carroll RW (1988). Relative density, range extension, and conservation potential of the lowland gorilla *(Gorilla gorilla)* in the Dzanga-Sangha region of southwestern Central African Republic. Mammalia.

[pbio-0030371-b07] Fay M, Agnagna M (1992). Census of gorillas in northern Republic of Congo. Am J Primatol.

[pbio-0030371-b08] Stromayer AK, Ekobo A (1992). The distribution and number of forest dwelling elephants in extreme southeastern Cameroon. Pachyderm.

[pbio-0030371-b09] Williamson E, Usongo L (1996). Gorilla survey in the Dja Reserve, Cameroun. Gor Conserv News.

[pbio-0030371-b10] Bermejo M (1999). Status and conservation of primates in Odzala National Park, Democratic Republic of Congo. Oryx.

[pbio-0030371-b11] Monath TP (1999). Ecology of Marburg and Ebola viruses: Speculations and directions for future research. J Infect Dis.

[pbio-0030371-b12] Rodriguez LL, De Roo A, Guimard Y, Trappier SG, Sanchez A (1999). Persistence and genetic stability of Ebola virus during the outbreak in Kikwit, Democratic Republic of Congo, 1995. J Infect Dis.

[pbio-0030371-b13] Peterson AT, Bauer JT, Mills JN (2004). Ecologic and geographic distribution of filovirus disease. Emerg Infect Dis.

[pbio-0030371-b14] Peterson AT, Carroll DS, Mills JN, Johnson KM (2004). Potential mammalian filovirus reservoirs. Emerg Infect Dis.

[pbio-0030371-b15] Morvan JM, Deubel V, Gounon P, Nakoune E, Barriere P (1999). Identification of Ebola virus sequences present as RNA or DNA in organs of terrestrial small mammals of the Central African Republic. Microbes Infect.

[pbio-0030371-b16] Gonzalez JP, Nakoune E, Slenczka W, Vidal P, Morvan JM (2000). Ebola and Marburg virus antibody prevalence in selected populations of the Central African Republic. Microbes Infect.

[pbio-0030371-b17] Leroy EM, Telfer P, Kumulungui B, Yaba P, Rouquet P (2004). A serological survey of Ebola virus infection in central African nonhuman primates. J Infect Dis.

[pbio-0030371-b18] Rouquet P, Froment JM, Bermejo M, Kilbourn A, Karesh W (2005). Wild animal mortality monitoring and human Ebola outbreaks, Gabon and Democratic Republic of Congo, 2001–2003. Emerg Infect Dis.

[pbio-0030371-b19] Allela L, Bourry O, Pouillot R, Delicat A, Yaba P (2005). Ebola virus antibody prevalence in dogs and human risk. Emerg Infect Dis.

[pbio-0030371-b20] Tucker CJ, Wilson JM, Mahoney R, Anyamba A, Linthicum K (2002). Climatic and ecological context of the 1994–1996 Ebola outbreaks. Photogramm Eng Rem S.

[pbio-0030371-b21] Pinzon JE, Wilson JM, Tucker CJ, Arthur R, Jahrling PB (2004). Trigger events: Enviroclimatic coupling of Ebola hemorrhagic fever outbreaks. Am J Trop Med Hyg.

[pbio-0030371-b22] Holmes EC (2004). The phylogeography of human viruses. Mol Ecol.

[pbio-0030371-b23] Grenfell BT, Pybus OG, Gog JR, Wood JL, Daly JM (2004). Unifying the epidemiological and evolutionary dynamics of pathogens. Science.

[pbio-0030371-b24] Real LA, Henderson JC, Biek R, Snaman J, Lambert JT (2005). Unifying the spatial population dynamics and molecular evolution of epidemic rabies virus. Proc Natl Acad Sci U S A.

[pbio-0030371-b25] Smith DB, Pathirana S, Davidson F, Lawlor E, Power J (1997). The origin of hepatitis C virus genotypes. J Gen Virol.

[pbio-0030371-b26] Epperson BK (2003). Geographical genetics.

[pbio-0030371-b27] Yang Z, Bielawski JP (2000). Statistical methods for detecting molecular adaptation. Trends Ecol Evol.

[pbio-0030371-b28] Bourhy H, Kissi B, Audry L, Smreczak M, Sadkowska-Todys M (1999). Ecology and evolution of rabies virus in Europe. J Gen Virol.

[pbio-0030371-b29] Mondet B (2001). Yellow fever epidemiology in Brazil—New considerations. Bull Soc Pathol Exot.

[pbio-0030371-b30] Smith DL, Lucey B, Waller LA, Childs JE, Real LA (2002). Predicting the spatial dynamics of rabies epidemics on heterogeneous landscapes. Proc Natl Acad Sci U S A.

[pbio-0030371-b31] McCormick JB (2004). Ebola virus ecology. J Infect Dis.

[pbio-0030371-b32] Sullivan NJ, Geisbert TW, Geisbert JB, Xu L, Yang ZY (2003). Accelerated vaccination for Ebola virus haemorrhagic fever in nonhuman primates. Nature.

[pbio-0030371-b33] Garbutt M, Liebscher R, Wahl-Jensen V, Jones S, Moller P (2004). Properties of replication-competent vesicular stomatitis virus vectors expressing glycoproteins of filoviruses and arenaviruses. J Virol.

[pbio-0030371-b34] Jones SM, Feldmann H, Stroher U, Geisbert JB, Fernando L (2005). Live attenuated recombinant vaccine protects nonhuman primates against Ebola and Marburg viruses. Nat Med.

[pbio-0030371-b35] Brochier B, Costy F, Pastoret PP (1995). Elimination of fox rabies from Belgium using a recombinant vaccinia-rabies vaccine—An update. Vet Microbiol.

[pbio-0030371-b36] Sanchez A, Trappier SG, Mahy BWJ, Peters CJ, Nichol S (1996). The virion glycoproteins of Ebola viruses are encoded in two reading frames and expressed through transcriptional editing. Proc Natl Acad Sci U S A.

[pbio-0030371-b37] Suzuki Y, Gojobori T (1997). The origin and evolution of Ebola and Marburg viruses. Mol Biol Evol.

[pbio-0030371-b38] Swofford DL (2002). PAUP: Phylogenetic analysis using parsimony, version 4 [computer program].

[pbio-0030371-b39] Posada D, Crandall KA (1998). MODELTEST: Testing the model of DNA substitution. Bioinformatics.

[pbio-0030371-b40] Posada D, Buckley T (2004). Model selection and model averaging in phylogenetics: Advantages of Akaike information criterion and Bayesian approaches over likelihood ratio tests. Syst Biol.

[pbio-0030371-b41] Rambaut A (2000). Estimating the rate of molecular evolution: Incorporating non-contemporaneous sequences into maximum likelihood phylogenies. Bioinformatics.

[pbio-0030371-b42] Drummond AJ, Rambaut A (2003). BEAST, version 1.03 [computer program].

[pbio-0030371-b43] Yang Z (1997). PAML: A program package for phylogenetic analysis by maximum likelihood. Comput Appl Biosci.

[pbio-0030371-b44] Sheridan I, Pybus OG, Holmes EC, Klenerman P (2004). High-resolution analysis of hepatitis C virus adaptation and its relationship to disease progression. J Virol.

[pbio-0030371-b45] Shackelton LA, Parrish CR, Truyen U, Holmes EC (2005). High rate of viral evolution associated with the emergence of carnivore parvovirus. Proc Natl Acad Sci U S A.

[pbio-0030371-b46] Yang Z, Nielsen R, Goldman N, Pedersen AM (2000). Codon-substitution models for heterogeneous selection pressure at amino acid sites. Genetics.

[pbio-0030371-b47] Yang ZH, Wong WSW, Nielsen R (2005). Bayes empirical Bayes inference of amino acid sites under positive selection. Mol Biol Evol.

