# Supplementary material for: Wave-Like Spread of Ebola Zaire
Source: PLoS Biol. 2005 Oct 25;3(11):e371. doi: 10.1371/journal.pbio.0030371 (PMC1262627; doi:10.1371/journal.pbio.0030371)
Supplement: Supplementary file 1 [file pbio.0030371.sd001.pdf]

S IV. Simulating Spatial Spread

Our analyses of the spatio-temporal pattern of ZEBOV outbreaks suggested a rate of spread that was consistent amongst major legs of the proposed epizootic. Genetic divergence and spatial separation were also correlated, both in a pairwise analysis of Gabon-Congo border sites separated by a maximum of about 120km and when genetic divergence and spatial separation were measured from the earliest outbreak at Yambuku, a distance of up to 1,400km. An interesting question is, then, how likely this kind of cross-scale spatial structuring is under the hypothesis of recent spread. An important corollary is how likely it is that such structure would be detectable given the small samples operative in our study.

We used a latticed based simulation model representing viral evolution in a spreading epizootic to address these questions. Although we tried to construct the model so that it would bear some similarity to our empirical system, our objective was not to perfectly reproduce the behavior of our system but rather explore the general tendency for spreading populations to produce cross scale structure that is detectable with small sample sizes.

TheModel

The simulation model was run on a rectangular lattice, with a single genome located at each node of the lattice. Each genome consisted of 1000 “sites” that assumed the binary states (0,1). At each step of a given simulation, the state at each site in each genome mutated randomly and independently between states with probability μ. At each time step with probability δ, each genome sent a propagule to replace the genome in one of

its four immediately adjacent neighbors. All directions (up, down, left, right) were equally likely to receive the propagule. The boundaries of the grid were absorbing so that a genome could send propagules off the lattice but there was no dispersal back onto the lattice. This stepping stone model of dispersal was chosen because the very tight spatial structure observed in our small scale analysis of ZEBOV-GP and the continuation of spatial structure at higher spatial scales strongly implies short distances between Ebola transmission events.

The spread simulations started with the infection of a cluster of ten nodes at the middle of one end of the lattice. All of these initial genomes were identical with all sites set to state “0”. Nodes on the rest of the lattice were initially empty but could be populated by dispersal from adjacent cells. Once occupied, at each time step a given node became “inactivated” with a fixed probability which we will hereafter refer to in terms of its complement, the persistence probability. Once inactivated, a node could neither mutate nor receive a dispersing propagule. This inactivation was intended to represent a decrease in the local density of susceptible hosts below the level necessary to sustain the virus, either through the development of host immunity or through host mortality. We performed runs of the simulation with the persistence probability set to both higher (0.99) and lower (0.95, 0.9) values.

We ran simulations using either a long, narrow lattice (10x1000nodes) or a wider lattice (100x1000nodes). Within each simulation run migration probability was set to either high (0.9), medium (0.7), or low (0.5). With these migration rates, the number of time steps necessary for the epizootic to transit the 1,000 node lattice ranged from 2,500 to

4,500. One caveat is that with low migration probability and low persistence probability the epizootic tended to burn out before it reached the end of the lattice. Results from these simulations were, therefore, discarded. We set the mutation probability to 0.00002 mutations/site/timestep which, given the number of time steps necessary to transit the lattice, produced total divergences roughly equivalent to that observed in the real ZEBOV sequences.

We sampled the spread pattern for simulation output in two ways. First, we chose nodes systematically distributed at 100node intervals across the middle of the lattice and recorded when the spreading wave front first reached each node. We used standard linear regression to estimate the slope of the relationship between distance from the origin and time at first arrival. We used the coefficient of determination (R^2) for the Pearson Product Moment Correlation to measure correlation strength. We then cut the lattice into non-overlapping partitions of 100 nodes and recorded the time of first arrival for each of ten sites distributed at 10 node intervals along the horizontal (long) axis of the lattice. We estimated the regression slope and the coefficient of determination separately for each partition and compared these to the results for the entire lattice.

We used a similar approach to sample the relationship between genetic divergence and spatial separation, except in this case we attempted to make the sample sizes and relative position of samples on the lattice more similar to those for ZEBOV. Thus, for the large scale pattern we sampled eleven nodes, with the sampling locations corresponding roughly to the relative distances of the real outbreaks from Yambuku. To add an element of stochasticity, we was chose the nodes randomly from a 50node

neighborhood centered on their expected positions. We then regressed the genetic divergence of each node from the genome used to initiate each simulation against both the time step at epizootic arrival and the distance from the origin. Within each of the ten 100node partitions of the lattice, we performed pairwise correlations of genetic divergence and spatial separation using six nodes distributed diagonally across the lattice. Although we present results from only one replicate for each choice of parameters and domain size, additional replicates produced highly similar results under all the simulation conditions.

Results

The spread simulations showed very consistent spread patterns. This was most evident when migration probability was high (0.9), local persistence probability was high (0.99) and the lattice narrow (10x1000 nodes). Under these conditions the "epizootic" moved smoothly across the lattice at a highly linear rate with very little variance (Fig. S4a). The very consistent rate of movement meant that spread was detectable on a local scale with relatively small sample sizes. For instance, when the simulation lattice was cut into partitions of 100nodes and ten samples were spaced ten nodes apart across each partition, all partitions showed correlation coefficients of determination of 0.99 (all highly significant). All of the partitions also showed spread rates that were very similar to the mean spread rate for the entire lattice (Fig. S4b). Simply expanding the lattice to 100x1000nodes had virtually no impact on the consistency of spread rate. Decreasing the migration probability to 0.7 or 0.5 and decreasing local persistence probability to 0.95 increased the probability of local "burnout" of the epizootic, thereby, creating a slightly more jagged wave front. However, the large scale pattern of spread was still

very strong with and all of the local neighborhoods still showed coefficients of determination higher than 0.96.

Because spread rate on the long narrow (10x1000 nodes) lattice was so consistent and genetic evolution was by assumption clocklike, genetic divergence of the wave front from the initial genome showed highly linear relationships with both spatial distance from the origin and time after the start of the epizootic (Fig. S5a). The very low variance in these relationships ensured a strong large scale correlation between genetic divergence and spatial separation, even with relatively small samples sizes comparable to those available for ZEBOV.

Increasing the height of the simulation domain to 100x1000nodes did not substantively alter the central tendency towards a roughly linear increase of genetic divergence with increasing spatial distance. However, it did increase the variance of this relationship (Fig. S5b). Because the wave front now included a larger population of genomes, different portions of the front were able to genetically "drift" away from each other as well as away from the initial genome at the origin. Migration along the wave front also occasionally brought moderately divergent lineages into close spatial proximity, creating variance at the low end of the distance scale. The high consistency of spread rate also produced a clocklike divergence of genotypes at the front of the wave from the original genotype (Fig. S5c).

Because the genetic-geographic divergence relationship was so linear and had so little variance, spatial structuring of genotypes was easily detectable at smaller scales. For the narrow lattice with high migration rate and high persistence, eighty percent of small scale (100node) partitions of the lattice showed significant correlations between spatial distance from the origin and genetic divergence (shown as Coefficient of

Determination in Fig. S6) when the same sample size as for ZEBOV was used (i.e. 6 samples). About one quarter of partitions produced correlations strengths of approximately the same magnitude as the correlation strength observed in the actual small scale correlations for ZEBOV (i.e. R^2=0.7). Strong correlations tend to be clustered near the origin, suggesting that later in the spread process the presence of divergent lineages in the same neighborhood tended to mask local isolation by distance within closely related lineages.

Increasing lattice width, decreasing migration probability, and decreasing persistence probability tended to decrease correlation strength for small scale genetic divergence by spatial separation regressions. However, small scale genetic structure was still detectable with small sample sizes under a wide range of simulation conditions. Varying mutation rate did not qualitatively change the central tendency towards roughly linear genetic divergence in time and space. However, it did affect statistical power, making it more difficult to detect spatial structure at small spatial scales.

In summary, the simulations showed that a simple nearest neighbor contact process can produce spread rates that were highly linear and similar at small and large scales and detectable with small sample sizes. Genetic divergence was also linear in time and space and, like spatial spread, could be detected with small sample sizes. Enlarging the simulation domain or decreasing persistence probability weakened genetic divergence correlations, but they were still consistent across scales and detectable with small sample sizes.

10x1000 nodes

A)

B)

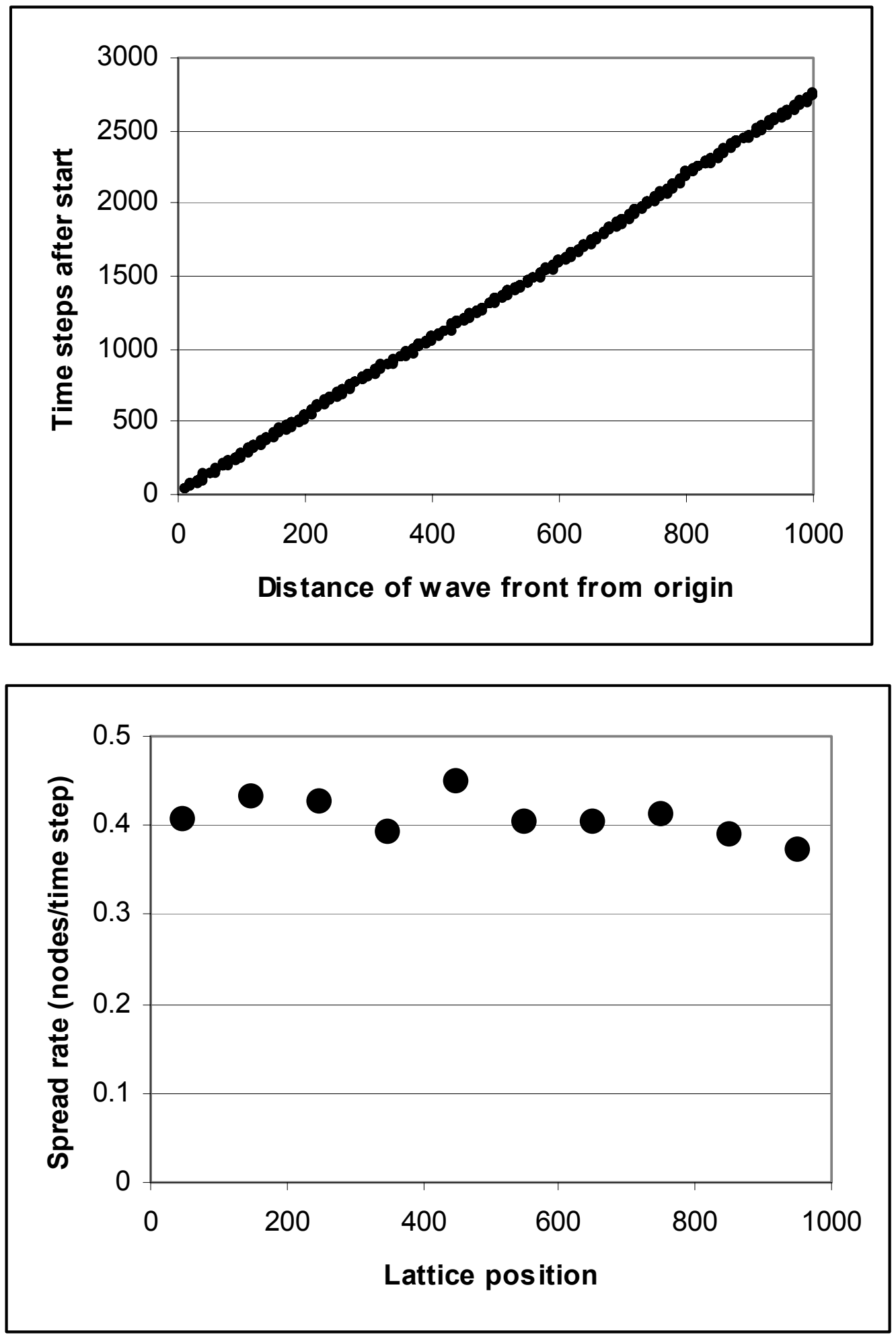

Figure S4: Epizootic spread rate. **A)** Pattern of spread on 10x1000 lattice with migration probability=0.9 and persistence probability=0.99. **B)** Slope of spread rate for samples of ten from 100node partitions of the lattice. None of the slopes for separate partitions fall far from the overall mean spread rate.

10x1000 nodes

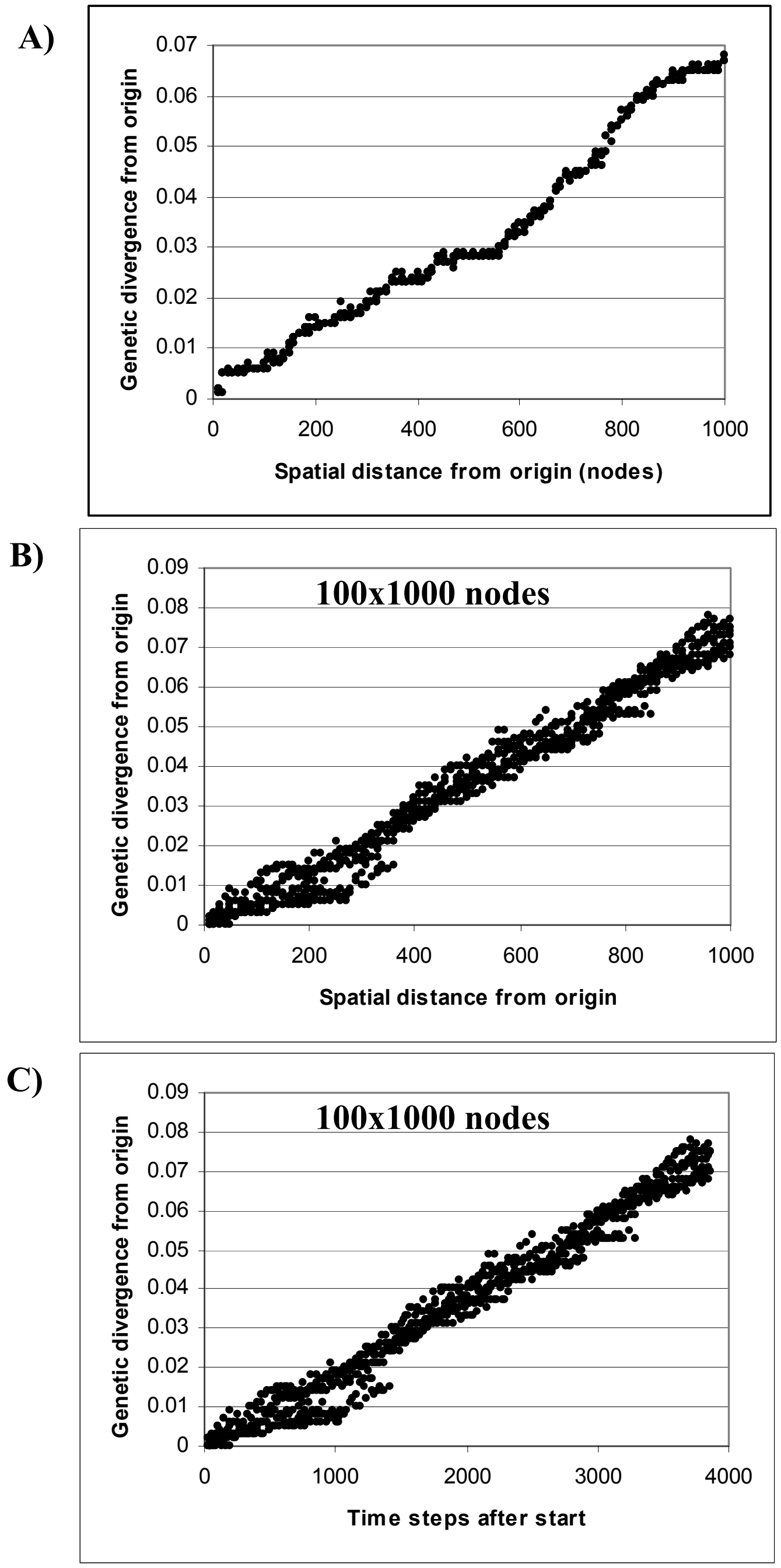

Figure S5: Large scale genetic divergence. **A)** Linear isolation by distance on narrow lattice using migration probability=0.9 and persistence probability=0.99. **B)** Reducing lattice width to 100nodes and migration probability to 0.5 increases local variance but maintains linearity of divergence. **C)** Clocklike genetic divergence with increasing time (same parameter values as B).

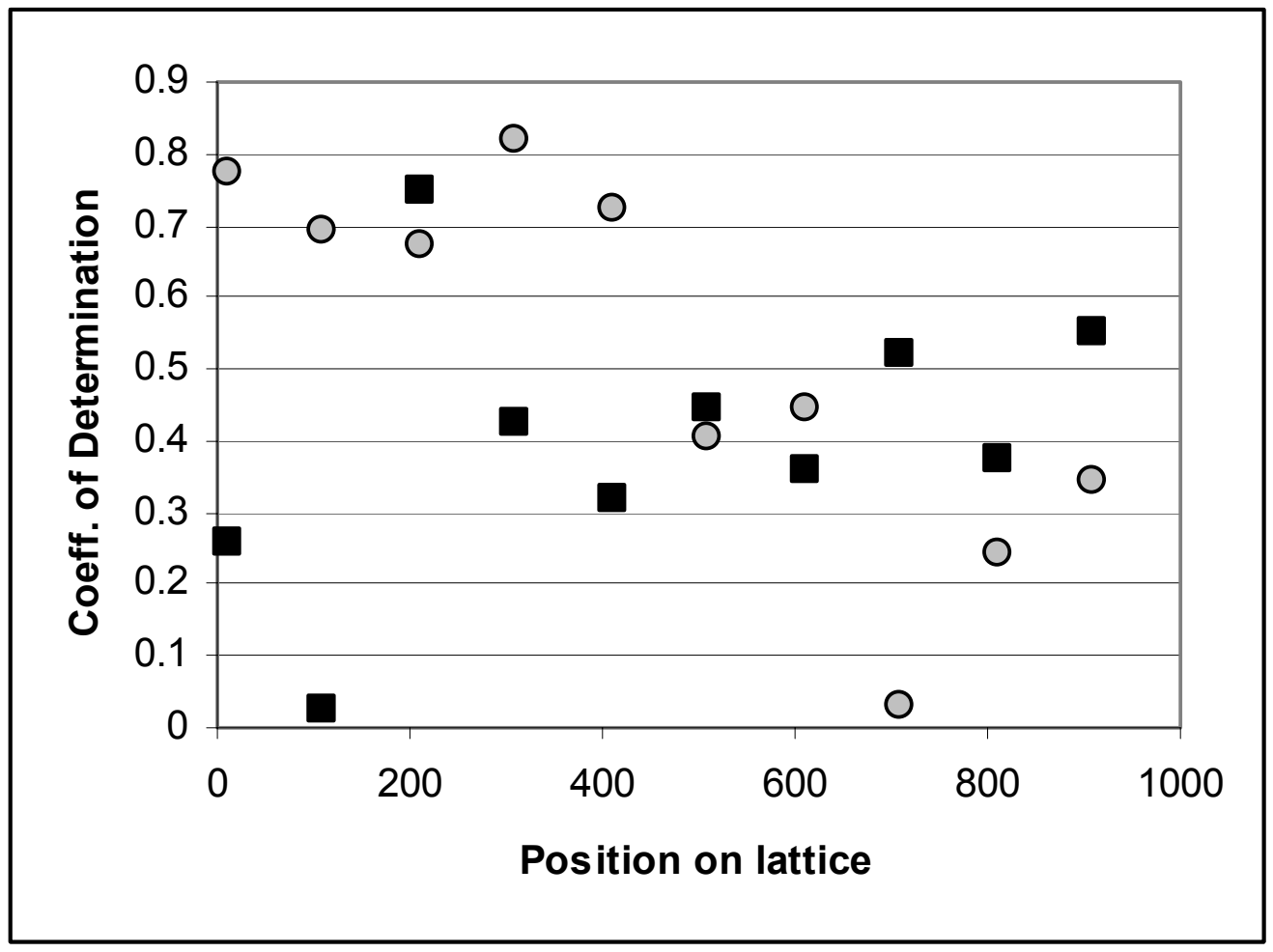

Figure 6: Small scale isolation by distance. Coefficient of determination for pairwise correlation of genetic divergence and spatial separation. Each point represents results for six sample sites drawn from within the same 100node local neighborhood. Results for two simulation runs are shown. Coefficient of determination (R^2) for real ZEBOV sequence data (n=6 samples) was 0.7. Values greater than 0.26 indicate statistically significant ($p<0.05$) local neighborhood correlation between genetic divergence and spatial separation.
