# Supplementary material for: Wave-Like Spread of Ebola Zaire
Source: PLoS Biol. 2005 Oct 25;3(11):e371. doi: 10.1371/journal.pbio.0030371 (PMC1262627; doi:10.1371/journal.pbio.0030371)
Supplement: Supplementary file 2 [file pbio.0030371.sd002.pdf]

**S II. Statistical Power Analysis**

One of the primary arguments against epizootic spread has been the apparent lack of GP sequence variation within human transmission chains which might imply a mutation rate too low to have generated the observed sequence variation during an epizootic lasting only a decade. Such variation, it is presumed, could only have accumulated through persistent, enzootic circulation of Ebola in some abundant reservoir species over a much longer time interval[2].

The shortcoming of this argument is that the sample of human transmission events available for estimating Ebola Zaire mutation rate is very small. Published GP sequences coming from transmission chains from which genetic data is available for all individuals involve a total of only 40 humans, including twenty seven from the recent Gabon-Congo border outbreaks[2] and thirteen from the Kikwit outbreak of 1995[14]. The probability of observing no mutations in forty transmission events can be estimated as

$$p( 0 \mid \mu, 40) = (1-\mu)^{40},$$

where $\mu$ is the mutation rate (i.e. the number of mutations per host generation). What this calculation reveals is that the available sample of Ebola Zaire transmission events is large enough to exclude with 95% confidence only a very high mutation rate. For example, the

probability of observing no mutations in the forty documented host to host transmission events, given a mutation rate of one mutation in fifty host generations, is

$$p( 0 \mid 1/50, 40) = (1-40)^{50} = 0.45.$$

The highest mutation rate that can be rejected with 95% confidence is one mutation per fifteen host generations,

$$p(0 \mid 1/15, 13) = (1\text{-}13)^{40} = 0.041.$$

Furthermore, some of the observed transmission chains for Ebola Zaire terminate in transmission from one human to several other humans. Each of these cases should be treated as one, not several host generations. Consequently the observed sample of host generations is actually lower than forty and the maximum mutation rate that can be excluded with 95% confidence is even higher than 1/13.

More generally, using the mutation rate observed in human transmission chains does not seem like a promising strategy for discriminating between enzootic and epizootic hypotheses. Without specifying exactly which species were involved in an Ebola Zaire epizootic and what proportion of individuals were infected, it is impossible to accurately estimate exactly how many host transmission generations might be involved. However, given the spatial scales over which there is evidence of Ebola Zaire impact in Gabon and Congo (tens of thousands of square kilometers) and the spatial densities typical of potential host species (ranging from a few per square kilometer for apes up to hundreds or thousands per square kilometer for small mammals), it is very reasonable to assume that thousands or, even, tens of thousands of hosts could have been infected. Consequently, even with mutation rates as low as 1/100 hosts or 1/1000 hosts, a large number of mutations could have accumulated in the GP gene during an epizootic lasting a few years. Excluding such mutation rates with 95% confidence would require transmission event samples many times larger than are currently available. This is apparent in Fig. S2, which for three different mutation rates (1/10, 1/100, and 1/1000) plots the probability of observing no mutations as a function of the number of transmission events observed. Excluding even lower mutation rates (i.e. rates too low to generate the observed level of sequence variation in an epizootic, given reasonable assumptions about the number of infected hosts) might require sample sizes on the order of thousands or tens of thousands.

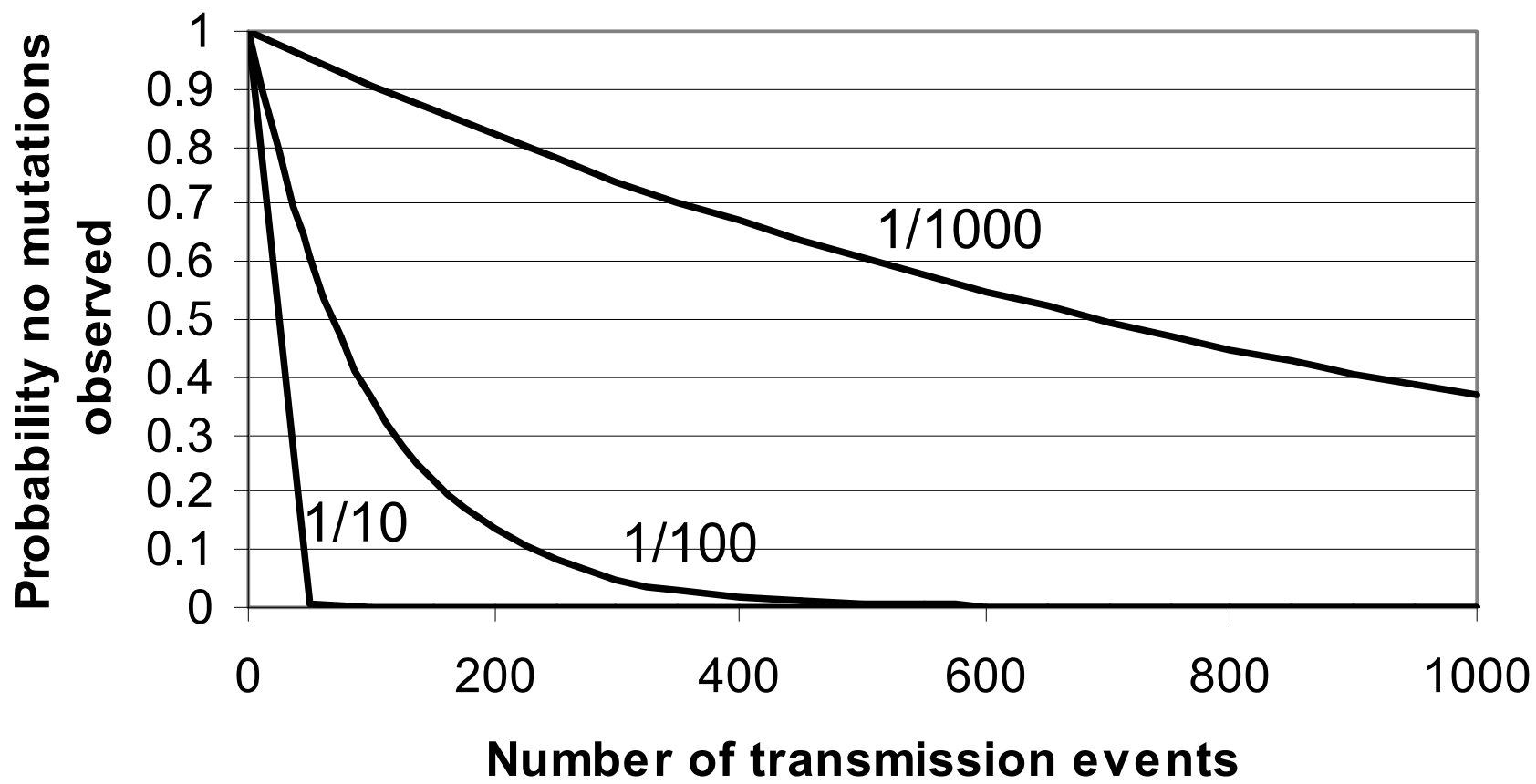


**Figure S2:** Sample sizes necessary to detect particular mutation rates.

## Additional Reference

S1. Thompson, J. D., Higgins, D. G. & Gibson, T. J. ClustalW: improving the sensitivity of progressive multiple sequence alignment through sequence weighting, position-specific gap penalties and weight matrix choice. Nucleic Acids Research 22, 4673-4680 (1994).
