# Supplementary material for: Wave-Like Spread of Ebola Zaire
Source: PLoS Biol. 2005 Oct 25;3(11):e371. doi: 10.1371/journal.pbio.0030371 (PMC1262627; doi:10.1371/journal.pbio.0030371)
Supplement: Supplementary file 3 [file pbio.0030371.sd003.pdf]

Supplementary Information

S I. Phylogenetic Estimation

Our phylogenetic analyses included thirteen of the fourteen published Zaire Ebolavirus (ZEBOV) GP gene sequences. We did not include the remaining published ZEBOV sequence, Yembelengoye '03 from northwest Congo, in any of our analyses because only a partial sequence (half as long as the other sequences) was available. Including it would have required throwing out half of the data for the other outbreak sites, thereby, greatly reducing the resolution of the analyses. We also excluded the Makokou sequence from our spatial regression analyses because, unlike the other sequences, the spatial origin of infection was not well localized. It came from a patient who was infected at an unknown site in the Gabon-Congo border region and then traveled more than 100km to the regional hospital at Makokou[2]. Thus, it did not provide usable information about the spatial hypotheses tested here. The Makokou sequence was, however, included in all phylogenetic analyses.

To establish ancestral relationships within ZEBOV, the ebolavirus sequence from Ivory Coast (ICEBOV), which previous analyses had identified as the closest known relative of ZEBOV[7,8], was included as an outgroup. Sequence alignment with ZEBOV sequences was performed using the Clustal W algorithm[S1]. While homology with ICEBOV could be established for much of the GP gene, alignment was uncertain for a variable region (positions 925-1500), which was therefore excluded for analyses involving ICEBOV (all alignments and phylogenies available from the authors on request). A suitable model of molecular evolution for ZEBOV was found using model averaging based on a 95% confidence set of models as implemented in Modeltest 3.6[28,29]. Based on this model (GTR+G), a ML tree was obtained in PAUP* v4.0.b10[27] under the heuristic search option with tree bisection-reconnection. Node support was evaluated through 1000 bootstrap replicates (Fig. S1). Because of the large divergence

of ICEBOV from ZEBOV, evolutionary model and phylogeny were subsequently re-estimated for the ZEBOV data only and using the entire length of the sequences, while retaining the established tree root. Methods were as described before. Branch lengths of the resulting ML tree (Fig. 2), estimated under a GTR + I model, where used in all subsequent analyses.

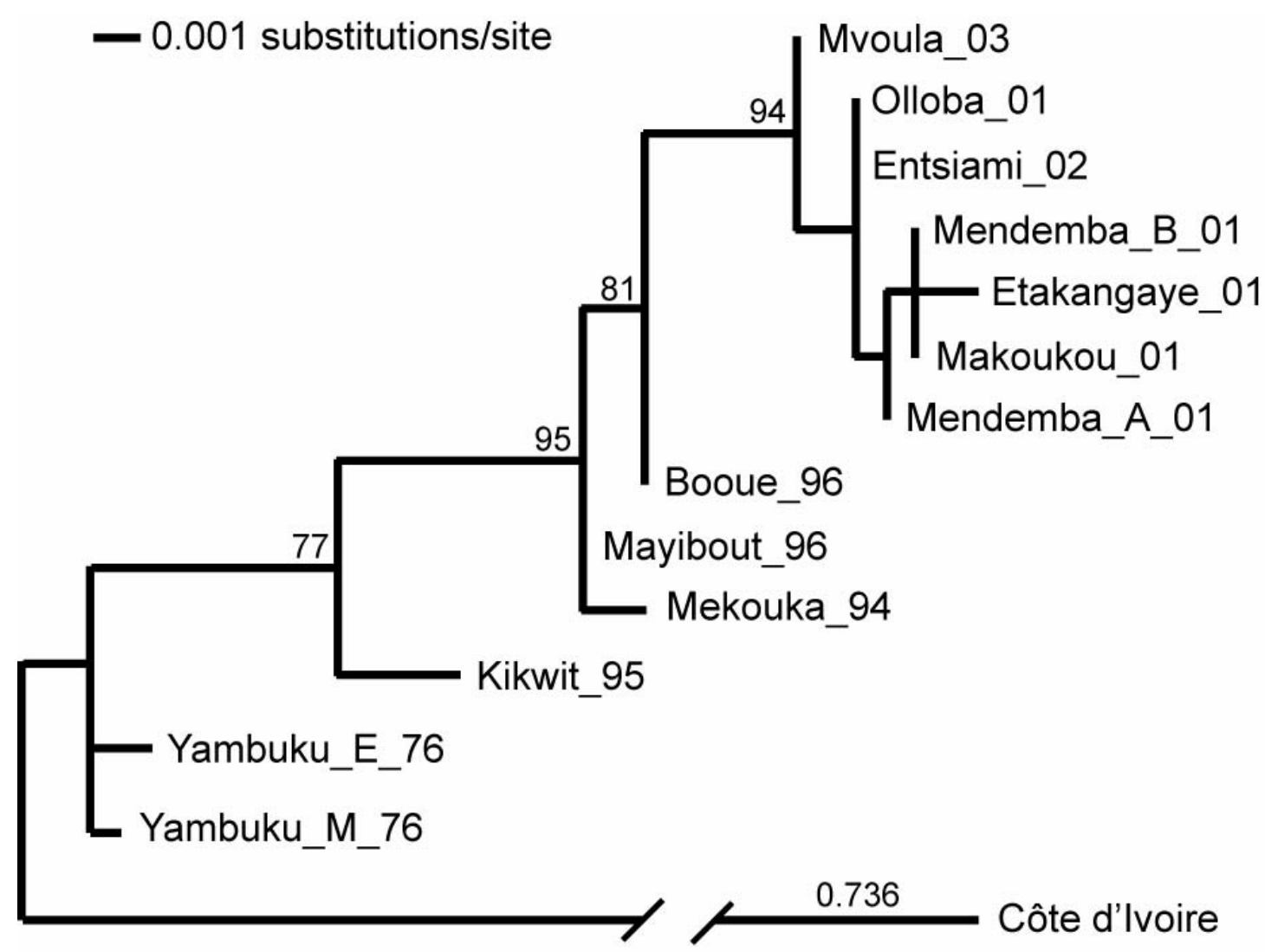

Figure S1

Table S1. Support for the molecular clock in ZEBOV. The single rate model (SR), representing a clock that ignores sampling dates, can be rejected in favour of the model allowing different rates for each branch (DR), whereas the single rate model with dated tips (SRDT) cannot be rejected.

Model	ln L	K[a]	Δ	d.f.	χ^2
DR	-3283.87	23	–	–	–
SR	-3315.12	12	31.26	11	P = 0.001
SRDT	-3300.94	13	17.07	10	P = 0.073

a = Number of estimated parameters

Table S2. . Assessing fit of selection models [43] to the ZEBOV glycoprotein data using likelihood ratio testing.

Model	ln L	Test	Δ	d.f.	χ^2
Site models					
M0 (constant)	-3221.64				
M1 (nearly neutral)	-3217.91				
M2 (positive)	-3213.25	M1 vs. M2	9.33	2	$P = 0.009$
M3 (discrete)	-3213.25	M0 vs. M3	16.79	4	$P = 0.002$
Branch models					
A. One ratio	-3221.64				
B. Two ratios[a]	-3221.28	A vs. B	0.36	1	$P = 0.395$

[a] specifies two classes of ratios, one for internal branches of the phylogeny, the other for external (i.e. tip) branches

Table S3. Evolutionary rate estimates for ZEBOV obtained under Maximum Likelihood (ML) in program TipDate and under a Bayesian framework using Markov Chain Monte Carlo (MCMC) integration in program BEAST.

Inference method	Rate [a]	95% CI or HPD [b]
ML	7.39×10^{-4}	4.03×10^{-4} – 1.03×10^{-3}
Bayesian MCMC	9.50×10^{-4}	5.78×10^{-4} – 1.36×10^{-3}

a = substitutions per site per year

b = confidence intervals or highest posterior densities
