# Supplementary material for: Wave-Like Spread of Ebola Zaire
Source: PLoS Biol. 2005 Oct 25;3(11):e371. doi: 10.1371/journal.pbio.0030371 (PMC1262627; doi:10.1371/journal.pbio.0030371)
Supplement: Supplementary file 4 [file pbio.0030371.st001.pdf]

Table S1. Support for the molecular clock in ZEBOV. The single rate model (SR), representing a clock that ignores sampling dates, can be rejected in favour of the model allowing different rates for each branch (DR), whereas the single rate model with dated tips (SRDT) cannot be rejected.

Model	ln L	K[a]	Δ	d.f.	χ^2
DR	-3283.87	23	–	–	–
SR	-3315.12	12	31.26	11	P = 0.001
SRDT	-3300.94	13	17.07	10	P = 0.073

a = Number of estimated parameters
