# Supplementary material for: Wave-Like Spread of Ebola Zaire
Source: PLoS Biol. 2005 Oct 25;3(11):e371. doi: 10.1371/journal.pbio.0030371 (PMC1262627; doi:10.1371/journal.pbio.0030371)
Supplement: Supplementary file 5 [file pbio.0030371.st002.pdf]

Table S2. . Assessing fit of selection models [43] to the ZEBOV glycoprotein data using likelihood ratio testing.

Model	ln L	Test	Δ	d.f.	χ^2
Site models					
M0 (constant)	-3221.64				
M1 (nearly neutral)	-3217.91				
M2 (positive)	-3213.25	M1 vs. M2	9.33	2	*P* = 0.009
M3 (discrete)	-3213.25	M0 vs. M3	16.79	4	*P* = 0.002
Branch models					
A. One ratio	-3221.64				
B. Two ratios[a]	-3221.28	A vs. B	0.36	1	*P* = 0.395

[a] specifies two classes of ratios, one for internal branches of the phylogeny, the other for external (i.e. tip) branches
