# Supplementary material for: Wave-Like Spread of Ebola Zaire
Source: PLoS Biol. 2005 Oct 25;3(11):e371. doi: 10.1371/journal.pbio.0030371 (PMC1262627; doi:10.1371/journal.pbio.0030371)
Supplement: Supplementary file 6 [file pbio.0030371.st003.pdf]

Table S3. Evolutionary rate estimates for ZEBOV obtained under Maximum Likelihood (ML) in program TipDate and under a Bayesian framework using Markov Chain Monte Carlo (MCMC) integration in program BEAST.

Inference method	Rate [a]	95% CI or HPD [b]
ML	7.39×10^{-4}	4.03×10^{-4} – 1.03×10^{-3}
Bayesian MCMC	9.50×10^{-4}	5.78×10^{-4} – 1.36×10^{-3}

a = substitutions per site per year

b = confidence intervals or highest posterior densities
