# Supplementary material for: Wave-Like Spread of Ebola Zaire
Source: PLoS Biol. 2005 Oct 25;3(11):e371. doi: 10.1371/journal.pbio.0030371 (PMC1262627; doi:10.1371/journal.pbio.0030371)
Supplement: Supplementary file 7 [file pbio.0030371.sg001.doc]

**Figure S1.**

**Selection Analysis**

Distribution of inferred amino acid changes on the ZEBOV phylogeny. Substitutions were estimated using ancestral reconstruction in program CodeML. Codon site 370, the only site identified as being under positive selection, is shown in blue.
